# Predictors of Distant Metastasis and Prognosis in Newly Diagnosed T1 Intrahepatic Cholangiocarcinoma

**DOI:** 10.1155/2023/6638755

**Published:** 2023-01-17

**Authors:** Kaibo Guo, Yidan Lou, Song Zheng

**Affiliations:** ^1^Department of Oncology, Affiliated Hangzhou First People's Hospital, Zhejiang University School of Medicine, Hangzhou 310006, China; ^2^Key Laboratory of Clinical Cancer Pharmacology and Toxicology Research of Zhejiang Province, Affiliated Hangzhou First People's Hospital, Zhejiang University School of Medicine, Hangzhou 310006, China; ^3^Zhejiang University School of Medicine, Hangzhou 310006, China; ^4^Department of Oncology, Affiliated Hangzhou Cancer Hospital, Zhejiang University School of Medicine, Hangzhou 310006, China

## Abstract

**Background:**

According to American Joint Committee on Cancer (AJCC) 8th staging system, T1 intrahepatic cholangiocarcinoma (T1 ICC) is considered a tumor with no vascular invasion. However, T1 ICC usually occurs distant metastasis (DM), and the clinical features of these patients could help clinicians identify the high-risk population.

**Methods:**

We reviewed 1959 newly diagnosed patients with T1 ICC from the Surveillance, Epidemiology, and End Results (SEER) database during 2004–2018. Logistic regression models and Cox proportional hazards models were conducted to predict the risk of DM and overall survival (OS), respectively, and then, web-based nomograms were constructed. Decision curve analysis (DCA) and clinical impact curves (CIC) were used to measure the clinical utility of the models. The low-, medium-, and high-risk groups were identified by calculating the summary of the risk points. Nomograms on the web were also created to help clinicians better use these prediction models.

**Results:**

Tumor size and lymph node metastasis accounted for the first two largest proportions among the DM nomogram scores, while surgery, DM, age at diagnosis, chemotherapy, and lymph node metastasis occupied the largest percentage in OS nomogram. DM nomogram was established for these newly diagnosed patients with T1 ICC, and OS nomogram was developed to visually predict the OS rate of 3, 5, and 10 years. The calibration curves revealed a valid predictive accuracy of nomograms, of which the C-index was 0.703 and 0.740, respectively, for good discrimination. DCAs, CICs, and risk subgroups showed the clinical validity of these nomograms. Two websites were created to make it easier to use these nomograms.

**Conclusions:**

Novel web-based nomograms predicting the risk of DM and OS for T1 ICC were constructed. These predictive tools might help clinicians make precise clinical strategies for each patient with T1 ICC.

## 1. Introduction

Cholangiocarcinoma is the second most common primary malignant tumor of the liver after hepatocellular carcinoma [1], and what occurs in the periphery of the liver, proximal to the second bile ducts, is called intrahepatic cholangiocarcinoma (ICC), which accounts for about 15%-20% of all primary liver cancers [2, 3]. ICC is regarded as an aggressive malignancy for poor prognosis with a 5-year overall survival (OS) of only around 9% [4]. Surgical resection in early stage might be the only potentially curative therapy. However, the recurrent disease occurred in about 60%-70% of patients [5–7], and even when patients undergo radical surgical treatment, 5-year OS still remains dismal at 20%-35% [8]. For patients with metastatic ICC, palliative care treatment is the main treatment with a median survival of 12.9 months [9].

There are increasing studies to identify the prognosis for ICC patients. Retrospective studies identified several parameters promoting poorer prognosis of ICC patients, including elder age, male, larger tumor size, higher grade, tumor-associated lymphangiogenesis, and advanced American Joint Committee on Cancer (AJCC) stage [10, 11]. According to the AJCC 8th staging system, T1 ICC has no vascular invasion. Interestingly, a part of T1 ICC patients usually show distant metastasis (DM), which is frequently associated with a poor prognosis and distinct clinical decision. Thus, the probability of DM needs to be taken into consideration in the management of treating patients with T1 ICC. With the increasing incidence of ICC worldwide in recent years, accurate prediction tools for these patients are in need, especially in early stage, although TNM staging system is the most common tool for predicting prognosis in patients of ICC. The TNM staging system does not include demographic characteristics such as age, gender, and race and cannot predict DM for those in early stage. Therefore, a more individual prediction for the probability of DM and the prognosis of T1 ICC patients is urgently necessary.

Herein, the objective of our study was to identify the risk factors for DM and identify prognostic factors of T1 ICC based on the Surveillance, Epidemiology, and End Results (SEER) database, which includes a large cohort of patients with detailed clinical information. Then, we aimed to establish novel web-based nomograms to predict the probability of DM and the OS rate of 3, 5, and 10 years in T1 ICC patients. With the guidance of these novel nomograms, management decisions for these patients will be optimized.

## 2. Methods

### 2.1. Data Extraction and Population Inclusion

**Figure 1:**
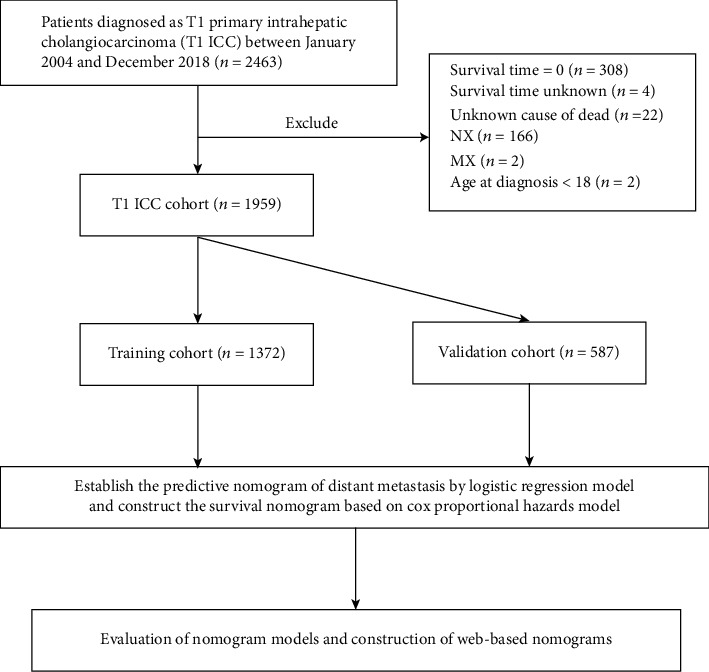
Analytical cohort and exclusion criteria of T1 ICC patients.

We obtained the data from the SEER 18 registry database (https://seer.cancer.gov/) by using SEER^∗^Stat 8.4.0 software. Based on the 2010 census, this public SEER database covers nearly 27.8% of the U.S. population. Cases of primary intrahepatic cholangiocarcinoma, diagnosed between January 2004 and December 2018, were identified by “The International Classification of Diseases for Oncology, 3rd Edition (ICD-O-3) Hist/behav, malignant” and “C22.1-Intrahepatic bile duct.” Cancer staging was integrated into the AJCC 8th edition, and T1 patients who participated in the study were involved in this study, who were subsequently randomized into two groups, including a training cohort and a validation cohort, in a ratio of 7 : 3. The screening flowchart is shown in Figure 1. Ethical approval is not necessary for this study, because the publicly available SEER database is composed of open-access and anonymous data.

### 2.2. Variable Exhibition and Outcomes

The following variables were collected, including year of diagnosis (2004-2008, 2009-2013, and 2014-2018), age at diagnosis (18-49, 50-64, 65-79, and ≥80 years), race (white, black, and other), sex (female and male), marital status (married and unmarried), tumor size (≤3 cm, 3-5 cm, 5-7 cm, 7-9 cm, >9 cm, and unknown), grade (I-II, III-IV, and unknown), lymph node metastasis (no and yes), DM (no and yes), radiation treatment (no/unknown and yes), chemotherapy (no/unknown and yes), surgery (no/unknown and yes), survival status (alive, dead of cancer, and dead of other), and follow-up time. The status of DM and OS was used as outcomes in this study, respectively.

### 2.3. Nomogram Development and Evaluation

Univariate and multivariable binary logistic regression models were constructed to identify the risk factors of DM in patients with T1 ICC. As for the OS of these patients, Cox proportional hazards models were used to find out the prognostic factors, while a competing risk model was used to estimate cancer-specific survival. Odds ratio (OR), hazard ratio (HR), subhazard ratio (SHR), and their 95% confidence interval (CI) were reported for the above factors. We developed nomograms based on these multivariable models for predicting the probability of DM and the OS of T1 ICC patients in the training cohort. Nomograms on the web were also created to help clinicians better use these prediction models. Furthermore, the low-, medium-, and high-risk groups were identified by calculating the summary of the risk points.

Calibration curves could assess the accuracy of the nomogram, and the concordance index (C-index) could quantify the discriminatory power of models. These two were usually used to validate the nomograms in both the training cohort and the validation cohort. Furthermore, decision curve analysis (DCA) and clinical impact curves (CIC) were conducted to calculate the clinical effectiveness of the nomograms.

### 2.4. Statistical Analysis

All data were analyzed using R software (version 3.6.1) with relevant packages and functions, such as rms, survival, and shinyPredict. *P* < 0.05 was considered statistically significant.

## 3. Results

### 3.1. Patient Enrollment and Characteristics

According to inclusion criteria, a total of 1959 T1 ICC patients were collected in the SEER database between 2004 and 2018. After randomization, there were 1372 patients in the training cohort and 587 patients in the validation cohort, respectively. The median age of the included patients was 68 (interquartile range, 59-76) years, including 962 (49.1%) females and 997 (50.9%) males. Among all patients, there were 1495 whites (76.3%), 170 blacks (8.7%), and 294 patients of other races (15.0%). The percentage of married patients was 57.0%, larger than unmarried. There were 352 (18.0%), 402 (20.5%), 299 (15.3%), 206 (10.5%), and 239 (12.2%) patients with ≤3 cm, 3-5 cm, 5-7 cm, 7-9 cm, and >9 cm, respectively, and tumor size of 461 patients was unknown. Concerning the status of lymph node metastasis and DM, a major proportion of patients (81.1%) were no lymph node metastasis, and 1470 (75.0%) patients showed no DM. In terms of treatment information, radiation treatment was performed for only 320 (16.3%) patients, and about half of patients (47.6%) received chemotherapy, while surgery was performed for 73.0% of the patients. The clinicopathological characteristics of T1 ICC patients are listed in Table 1.

### 3.2. Risk Factors of DM and Construction of the Nomogram

Risk factors for DM were identified by univariable and binary logistic regression analysis. The results of this study revealed that the significant independent risk factors for DM comprised age at diagnosis, tumor size, grade, and lymph node metastasis (Table 2). When the age was less than 80, older age showed a potentially higher risk for patients with T1 ICC to occur DM. Approximate to patients whose tumor size was less than 3 cm, those with 7-9 cm (OR = 2.46, 95% CI = 1.45 − 4.22, *P* = 0.001) and over 9 cm of tumor size (OR = 3.66, 95% CI = 2.21 − 6.14, *P* < 0.001) were at a higher risk of DM. Increasing risk of DM was found in patients with unknown grades (OR = 1.92, 95% CI = 1.37 − 2.72, *P* < 0.001). In addition, patients who suffered from lymph node metastasis showed an increased risk of DM (OR = 2.81, 95% CI = 2.09 − 3.77, *P* < 0.001).

**Figure 2:**
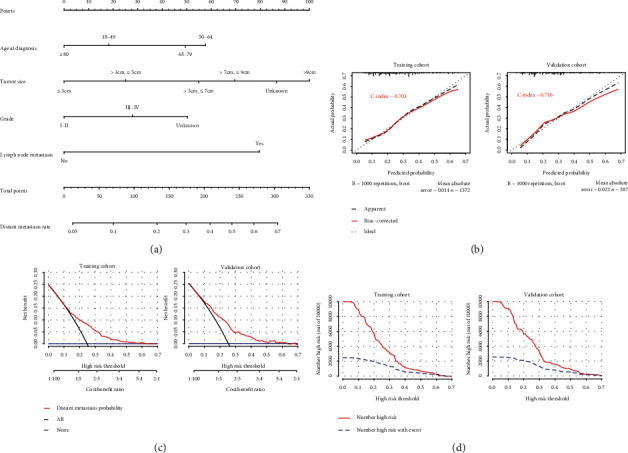
Nomogram, calibration curve, decision curve analysis, and clinical impact curve for predicting distant metastasis (DM) in patients with T1 ICC. There are four factors in DM prediction nomogram (a). Calibration curve (b) for predicting DM is shown, and C-index was 0.703 in the training cohort and 0.716 in the validation cohort. The decision curve (c) of the nomogram predicting DM was plotted. The *x*-axis represents the threshold probability and the *y*-axis represents the net benefit. The horizontal blue line represents one extreme situation that no patients suffered DM, and the black line represents that all patients experience DM. Clinical impact curve (d) shows that the number of high-risk patients and the number of high-risk patients with event were plotted by different threshold probability in a population.

To exhibit the risk factors for DM in T1 ICC, a nomogram model was developed (Figure 2). The length of the straight line for each variant in the nomogram (Figure 2(a)) indicated its contribution to the DM risk. Score assignments for each independent factor could be obtained in Table S1, and then, the DM result could be predicted by correlating functional conversions between the total scores and the predicted DM rate (Table S2). As was shown in the nomogram, tumor size accounted for the biggest value of contribution, followed by lymph node metastasis, age at diagnosis, and grade.

The calibration curves performed an effective predictive accuracy of the nomogram for predicting DM, with relatively high C-indexes in both the OS (0.703) and the validation cohort (0.716). The diagonal line showed that the actual and predicted DM probabilities are equal, and the solid line showed the actual observations. When the solid line was close to the diagonal line, the nomogram showed good agreement between the probabilities of the nomogram and the actual outcome (Figure 2(b)). Decision curve analysis (DCA) and clinical impact curve (CIC) of the nomogram (Figures 2(c) and 2(d)) indicated that a threshold probability of 0.1-0.6 was most favorable for the predictive ability of DM in both the training and the validation cohorts.

### 3.3. Prognostic Factors for T1 ICC and Establishment of the Nomogram

Prognostic factors for patients with T1 ICC were identified by univariable and multivariable Cox regression analyses (Table 3). We discovered that the following variables including age at diagnosis, race, tumor size, grade, lymph node metastasis, DM, radiation treatment, chemotherapy, and surgery were important factors for OS in T1 ICC patients. Compared with patients whose ages at diagnosis were 18-49 years, those aged 65-79 years (HR = 1.49, 95% CI = 1.17 − 1.90, *P* = 0.001) and aged over 80 (HR = 1.64, 95% CI = 1.24 − 2.16, *P* < 0.001) showed a higher risk of death. Patients of other races showed better prognosis than white patients (HR = 0.82, 95% CI = 0.68 − 0.98, *P* = 0.031). Patients who suffered lymph node metastasis had a significant death risk (HR = 1.44, 95% CI = 1.23 − 1.69, *P* < 0.001), and similar results occurred in distant metastasis (HR = 1.71, 95% CI = 1.47 − 1.99, *P* < 0.001). As for the association between the treatment and prognosis, the death probability often decreased when T1 ICC patients accepted surgical resection (HR = 0.29, 95% CI = 0.23 − 0.35, *P* < 0.001). Patients could OS benefit from radiation treatment (HR = 0.79, 95% CI = 0.67 − 0.94, *P* = 0.010) and chemotherapy (HR = 0.68, 95% CI = 0.59 − 0.78, *P* < 0.001), respectively. Moreover, we performed a competing risk model to determine the significant cancer-specific prognostic factors of T1 ICC, and the results were similar to the Cox model (Table 4).

**Figure 3:**
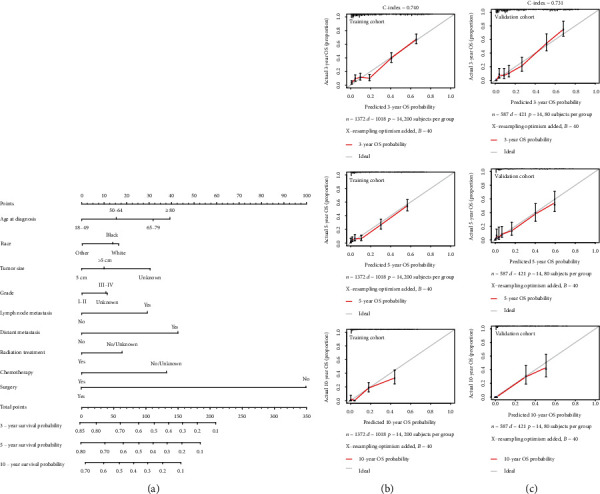
Nomogram and calibration curve for predicting overall survival (OS) in patients with T1 ICC. There are nine factors in OS prediction nomogram (a). Calibration curve for predicting 3-, 5-, and 10-year OS in the training cohort (b). Calibration curve for predicting 3-, 5-, and 10-year OS in the validation cohort (c).

Based on the Cox regression analysis, a nomogram was created to predict the probability of OS in T1 ICC (Figure 3). As was shown in the nomogram, surgery was the largest contribution, followed by DM, age at diagnosis, chemotherapy, tumor size, and lymph node metastasis, while radiation, race, and grade made little contribution. According to the effects on the survival outcomes, each clinicopathological variable had its point, which is listed in Table S3. Total points of prognostic factors could be used to predict 3-year, 5-year, and 10-year OS probabilities (Table S4-6).

The C-index of the nomogram was 0.740 in the training cohort and 0.731 in the validation cohort, which suggested that the nomogram could effectively predict the death risks in patients with T1 ICC. Calibration curves showed satisfactory agreement between the nomogram predictions and the actual observations of OS probabilities at 3, 5, and 10 years (Figures 3(b) and 3(c)). Moreover, DCA revealed that the nomogram for predicting 3-, 5-, and 10-year death probabilities would provide more net benefits compared to AJCC 8th staging and treatment.

### 3.4. Risk Stratification and Development of Web-Based Nomogram

**Figure 4:**
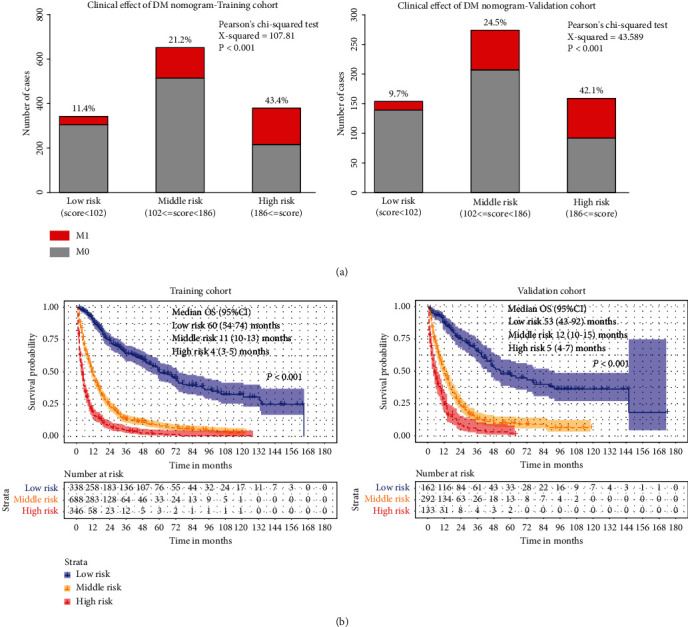
Clinical effects of the risk score in the nomogram. Based on the quartile of risk score, nomograms divided patients into low-, middle-, and high-risk subgroups, respectively. Clinical utility of these subgroups for predicting DM is presented by constituent ratio (a). The Kaplan-Meier method is used to find out the significance among these risk subgroups (b).

**Figure 5:**
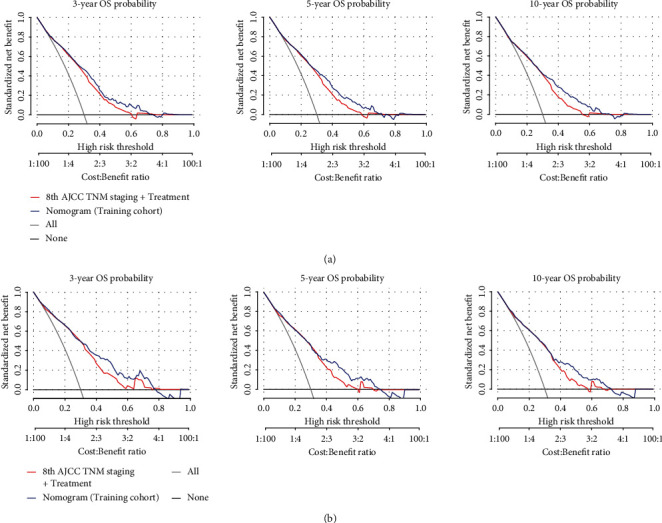
The decision curve of the nomogram predicting 3-, 5-, and 10-year OS in the training (a) and validation (b) cohorts was plotted. The *x*-axis represents the threshold probability, and the *y*-axis represents the standardized net benefit. The horizontal black line represents one extreme situation that all patients were alive, and the grey line represents the other extreme situation that all patients were dead.

To further investigate the clinical application of the nomograms, the total score for each patient was determined from the nomograms in both the training and the validation cohorts. According to the 25^th^ and 75^th^ percentile values of the risk scores (102 and 186 for DM nomogram, 128 and 232 for OS nomogram), the patients were divided into three risk levels: low-risk, middle-risk, and high-risk groups. The incidence of DM among these subgroups was significantly different in the training and the validation cohorts, with the high-risk group having a nearly 30% higher rate of DM than the low-risk group (Figure 4(a)). As far as OS, we used the Kaplan-Meier method, and the results showed that the OS of the low-, middle-, and high-risk groups in the training cohort and the validation cohort was significantly differentiated, with the median OS in the high-risk group being at least 4 years longer than in the low-risk group (Figure 4(b)). Moreover, our nomogram showed better results than 8th AJCC TNM staging in predicting 3-, 5-, and 10-year OS in both training cohort (Figure 5(a)) and validation cohort (Figure 5(b)).

To assist researchers and clinicians, our nomograms are available online. Online version of the T1-ICC distant metastasis nomogram (Figure S1) could be easily accessed at https://kaiboguo.shinyapps.io/T1ICCDMNomogram/, and online version of T1-ICC overall survival nomogram (Figure S2-4) could be accessed at https://kaiboguo.shinyapps.io/T1ICCOSNomogram/.

## 4. Discussion

According to AJCC 8th staging system, T1 stage is classified into T1a and T1b based on tumor diameter, neither of which has vascular invasion. However, there is heterogeneity in T1 ICC patients, and due to the highly aggressive biological characteristics, some patients have early DM, leading to poor prognosis [12]. Therefore, assessment and intervention in early stage are necessary. Furthermore, for T1 ICCs, patients with different stages have different treatment methods and prognosis because of distinct clinical features [13]. It is very important for patients with T1 ICC to distinguish the status of DM and to predict OS based on clinical-pathological characteristics.

ICC is relatively rare and compared to hepatocellular carcinoma, its staging systems are fewer [14]. With the increasing incidence of ICC, an accurate staging system is urgently needed. The AJCC staging system is now most commonly used in ICC patients, but this system has some limitations. First, it is more applicable to a broad population than to individuals based on the heterogeneity of T1 ICCs. Using AJCC staging to predict prognosis is sometimes too general to account for the diversity of treatments and individual patient outcomes. Currently, some nomograms have been developed for patients with ICC. Wang et al. constructed a nomogram to predict prognosis after partial hepatectomy [15]. Shen et al. developed a machine learning-based nomogram to identify ICC patients who were caused by intrahepatic lithiasis [16]. However, these nomograms do not reflect the probability of DM and OS in T1 ICC patients and are not applicable to those newly diagnosed.

In our study, in order to help clinicians better manage the patients with T1 ICC, we attempted to establish novel web-based nomograms for predicting the probability of DM and OS in these patients using readily available clinical data for the first time. DM nomogram included four factors: age at diagnosis, tumor size, grade, and lymph node metastasis, while OS nomogram for predicting 3-, 5-, and 10-year OS included nine factors: age at diagnosis, race, tumor size, grade, lymph node metastasis, DM, radiation treatment, chemotherapy, and surgery. Relatively high C-indexes of nomograms showed good accuracy of the models, and calibration curves in both training and validation cohorts showed good agreement between predictions and observations. Especially, DCA was performed to confirm that the nomograms gained additional net benefits compared with the AJCC 8th staging and treatment. Furthermore, according to the interquartile scores of the nomograms, we classified patients into low-, medium-, and high-risk groups and plotted stacked histograms and Kaplan-Meier survival curves, in which the discriminative power was confirmed.

In the population-based study, we found that a huge proportion of the DM nomogram scores accounted for tumor size over 9 cm, lymph node metastasis, and age 50 to 64 years. In this study, as a significant factor, the DM risk of tumor size over 9 cm and 7-9 cm rose to 3.66 and 2.46, respectively. One study revealed that the diameter and number of tumors were significantly associated with prognosis in multivariate analysis, and they represented the aggressiveness of the tumor [17]. However, few studies reported the relationship between DM of T1 ICC and its tumor size. N classification was an independent predictor of DM risk in T1 ICC, and patients occurring lymph node metastasis were prone to occur DM. OR of lymph node metastasis was up to 2.81, indicating the close association between lymph node metastasis and DM, which is not surprising that lymph node could be a way to occur DM [18]. In previous studies [19, 20], age was rarely used as a related variable for DM. However, this study found that the DM risk of patients aged 50 to 64 years was higher than other patients.

For OS nomogram, the largest percentage of risk scores was not undergoing surgery, DM, and age over 80 years old. Surgical resection for T1 ICC patients, as the only potentially curative therapy, has a survival benefit. Not surprisingly, patients with DM which were classified in stage IV showed poorer prognosis. In agreement with our results, reportedly older ICC patients are significantly associated with poorer prognosis [21]. In addition, there were other variables used in the OS nomogram, including lymph node metastasis, race, tumor size, grade, radiation treatment, and chemotherapy. Generally, the positive lymph node number is closely related to the prognosis for many malignancies, such as distal cholangiocarcinoma and gallbladder carcinoma, and a study has demonstrated that the prognosis of patients with ≥4 positive lymph nodes is similar to that of patients with DM [17]. As we know, only our study reported that race influences the OS of T1 ICC patients and patients with white and black experience worse prognosis. Besides, the cut-off value of the tumor size was controversial in different systems, and based on population-related survival analysis, the AJCC concluded that tumor size > 5 cm was correlated to poor prognosis [22], which is consistent with our findings. Higher grades also showed poorer prognosis, while radiation treatment and chemotherapy usually bring benefits to T1 ICC patients.

There were still some limitations in our study. Firstly, some studies [23, 24] suggested that serum CA199 and CEA levels are independent risk factors for prognosis of cholangiocarcinoma, which were not included as variables in our study because of the limited information retrieved from the SEER database. Secondly, the data from the public database lacked records of the distant site of metastasis. Finally, this study was a retrospective study including patients from the United States. Although our nomograms' internal validation showed good consistency, it still needs to be verified by external populations.

## 5. Conclusion

Two web-based nomograms conducted based on independent risk factors from a large public database could predict the distant metastasis and overall survival of T1 ICC patients. Furthermore, our nomograms have been validated by discrimination and calibration, showing a high degree of accuracy and reliability, as well as considerable clinical utility. Accordingly, with the guidance of the nomograms, more optimal decision-making will be undertaken to improve the prognosis of patients with T1 ICC.

## Figures and Tables

**Table 1:** Clinicopathological characteristics of patients with T1 intrahepatic cholangiocarcinoma.

Clinicopathological variables	Overall (*N* = 1959)	Training (*N* = 1372)	Validation (*N* = 587)	*P* value
Year of diagnosis				0.585
2004-2008	397 (20.3%)	286 (20.8%)	111 (18.9%)	
2009-2013	650 (33.2%)	455 (33.2%)	195 (33.2%)	
2014-2018	912 (46.6%)	631 (46.0%)	281 (47.9%)	
Age at diagnosis				0.293
18-49	158 (8.1%)	121 (8.8%)	37 (6.3%)	
50-64	637 (32.5%)	441 (32.1%)	196 (33.4%)	
65-79	845 (43.1%)	585 (42.6%)	260 (44.3%)	
≥80	319 (16.3%)	225 (16.4%)	94 (16.0%)	
Race				0.652
White	1495 (76.3%)	1055 (76.9%)	440 (75.0%)	
Black	170 (8.7%)	116 (8.5%)	54 (9.2%)	
Other	294 (15.0%)	201 (14.7%)	93 (15.8%)	
Sex				0.639
Female	962 (49.1%)	679 (49.5%)	283 (48.2%)	
Male	997 (50.9%)	693 (50.5%)	304 (51.8%)	
Marital status				0.42
Married	1116 (57.0%)	773 (56.3%)	343 (58.4%)	
Unmarried	843 (43.0%)	599 (43.7%)	244 (41.6%)	
Tumor size				0.024
≤3 cm	352 (18.0%)	239 (17.4%)	113 (19.3%)	
>3 cm, ≤5 cm	402 (20.5%)	295 (21.5%)	107 (18.2%)	
>5 cm, ≤7 cm	299 (15.3%)	188 (13.7%)	111 (18.9%)	
>7 cm, ≤9 cm	206 (10.5%)	153 (11.2%)	53 (9.0%)	
>9 cm	239 (12.2%)	165 (12.0%)	74 (12.6%)	
Unknown	461 (23.5%)	332 (24.2%)	129 (22.0%)	
Grade				0.267
I-II	532 (27.2%)	358 (26.1%)	174 (29.6%)	
III-IV	325 (16.6%)	232 (16.9%)	93 (15.8%)	
Unknown	1102 (56.3%)	782 (57.0%)	320 (54.5%)	
Lymph node metastasis				0.499
No	1580 (81.1%)	1107 (80.7%)	482 (82.1%)	
Yes	370 (18.9%)	265 (19.3%)	105 (17.9%)	
Distant metastasis	489 (25.0)	340 (24.8)	149 (25.4)	0.822
No	1470 (75.0%)	1032 (75.2%)	438 (74.6%)	
Yes	489 (25.0%)	340 (24.8%)	149 (25.4%)	
Radiation treatment				1.000
No/unknown	1639 (83.7%)	1148 (83.7%)	491 (83.6%)	
Yes	320 (16.3%)	224 (16.3%)	96 (16.4%)	
Chemotherapy				0.849
No/unknown	1017 (52.4%)	721 (52.6%)	305 (52.0%)	
Yes	933 (47.6%)	651 (47.4%)	282 (48.0%)	
Surgery				0.222
No/unknown	1430 (73.0%)	1013 (73.8%)	417 (71.0%)	
Yes	529 (27.0%)	359 (26.2%)	170 (29.0%)	
Survival status				0.227
Alive	520 (26.5%)	354 (25.8%)	166 (28.3%)	
Dead of cancer	1320 (67.4%)	940 (68.5%)	380 (64.7%)	
Dead of other	119 (6.1%)	78 (5.7%)	41 (7.0%)	
Follow-up time (months)	10 (4, 23)	9 (4, 23)	11 (4, 25)	0.219

**Table 2:** Logistic regression analysis of the risk factors for distant metastasis in patients with T1 intrahepatic cholangiocarcinoma.

Clinicopathological variables	Univariate analysis	*P* value	Multivariate analysis	*P* value
	OR (95% CI)		OR (95% CI)	
Age at diagnosis				
18-49	Reference		Reference	
50-64	1.52 (0.95-2.51)	0.092	1.67 (1.02-2.82)	0.049
65-79	1.35 (0.85-2.21)	0.22	1.50 (0.92-2.51)	0.112
≥80	0.78 (0.45-1.38)	0.387	0.79 (0.44-1.43)	0.43
Race				
White	Reference			
Black	1.03 (0.66-1.58)			
Other	0.80 (0.55-1.15)			
Sex				
Female	Reference			
Male	1.02 (0.80-1.30)			
Marital status				
Married	Reference			
Unmarried	0.98 (0.76-1.25)			
Tumor size				
≤3 cm	Reference		Reference	
>3 cm, ≤5 cm	1.40 (0.87-2.29)	0.17	1.38 (0.85-2.29)	0.197
>5 cm, ≤7 cm	1.99 (1.20-3.33)	0.008	2.04 (1.21-3.46)	0.008
>7 cm, ≤9 cm	2.80 (1.68-4.70)	<0.001	2.46 (1.45-4.22)	0.001
>9 cm	3.73 (2.30-6.18)	<0.001	3.66 (2.21-6.14)	<0.001
Unknown	3.37 (2.19-5.31)	<0.001	3.07 (1.97-4.91)	<0.001
Grade				
I-II	Reference		Reference	
III-IV	1.65 (1.09-2.49)	0.018	1.43 (0.93-2.21)	0.100
Unknown	2.10 (1.53-2.92)	<0.001	1.92 (1.37-2.72)	<0.001
Lymph node metastasis				
No	Reference		Reference	
Yes	3.01 (2.26-3.99)	<0.001	2.81 (2.09-3.77)	<0.001

**Table 3:** Cox regression analysis of the prognostic factors for OS in patients with T1 intrahepatic cholangiocarcinoma.

Clinicopathological variables	Univariate analysis	*P* value	Multivariate analysis	*P* value
	HR (95% CI)		HR (95% CI)	
Age at diagnosis				
18-49	Reference		Reference	
50-64	1.40 (1.09-1.80)	0.008	1.21 (0.94-1.56)	0.133
65-79	1.85 (1.45-2.36)	<0.001	1.49 (1.17-1.90)	0.001
≥80	2.71 (2.08-3.53)	<0.001	1.64 (1.24-2.16)	<0.001
Race				
White	Reference			
Black	0.98 (0.78-1.22)	0.833	0.97 (0.77-1.22)	0.784
Other	0.85 (0.70-1.01)	0.069	0.82 (0.68-0.98)	0.031
Sex				
Female	Reference			
Male	1.03 (0.91-1.17)	0.621		
Marital status				
Married	Reference			
Unmarried	1.09 (0.96-1.23)	0.175		
Tumor size				
≤5 cm	Reference		Reference	
>5 cm	1.2 (1.03-1.38)	0.017	1.13 (0.97-1.32)	0.105
Unknown	2.05 (1.76-2.40)	<0.001	1.47 (1.25-1.72)	<0.001
Grade				
I-II	Reference		Reference	
III-IV	1.47 (1.20-1.79)	<0.001	1.14 (0.93-1.40)	0.202
Unknown	2.21 (1.89-2.57)	<0.001	1.15 (0.97-1.36)	0.104
Lymph node metastasis				
No	Reference		Reference	
Yes	1.72 (1.48-2.00)	<0.001	1.44 (1.23-1.69)	<0.001
Distant metastasis				
No	Reference		Reference	
Yes	2.33 (2.02-2.67)	<0.001	1.71 (1.47-1.99)	<0.001
Radiation treatment				
No	Reference		Reference	
Yes	0.77 (0.65-0.92)	0.003	0.80 (0.67-0.95)	0.016
Chemotherapy				
No	Reference		Reference	
Yes	0.84 (0.74-0.95)	0.004	0.62 (0.54-0.72)	<0.001
Surgery				
No	Reference		Reference	
Yes	0.23 (0.19-0.27)	<0.001	0.29 (0.23-0.35)	<0.001

**Table 4:** Competing risk regression analysis of the OS in T1 intrahepatic cholangiocarcinoma.

Clinicopathological variables	Univariate analysis	*P* value	Multivariate analysis	*P* value
	SHR (95% CI)		SHR (95% CI)	
Age at diagnosis				
18-49	Reference		Reference	
50-64	1.27 (1.03-1.57)	0.027	1.10 (0.88-1.37)	0.400
65-79	1.47 (1.20-1.81)	<0.001	1.17 (0.95-1.45)	0.150
≥80	1.89 (1.47-2.42)	<0.001	1.13 (0.87-1.49)	0.360
Race				
White	Reference			
Black	1.038 (0.83-1.29)	0.740	1.00 (0.79-1.26)	1.000
Other	0.86 (0.71-1.03)	0.093	0.85 (0.70-1.03)	0.099
Sex				
Female	Reference			
Male	1.03 (0.91-1.17)	0.621		
Marital status				
Married	Reference			
Unmarried	1.05 (0.93-1.19)	0.44		
Tumor size				
≤5 cm	Reference		Reference	
>5 cm	1.30 (1.12-1.50)	<0.001	1.24 (1.06-1.46)	0.007
Unknown	1.97 (1.68-2.32)	<0.001	1.43 (1.20-1.71)	<0.001
Grade				
I-II	Reference		Reference	
III-IV	1.44 (1.18-1.76)	<0.001	1.13 (0.91-1.39)	0.280
Unknown	2.09 (1.80-2.43)	<0.001	1.18 (1.00-1.40)	0.057
Lymph node metastasis				
No	Reference		Reference	
Yes	1.64 (1.42-1.90)	<0.001	1.31 (1.10-1.55)	0.002
Distant metastasis				
No	Reference		Reference	
Yes	2.36 (2.07-2.70)	<0.001	1.74 (1.49-2.03)	<0.001
Radiation treatment				
No	Reference		Reference	
Yes	0.79 (0.68-0.92)	0.002	0.79 (0.67-0.94)	0.010
Chemotherapy				
No	Reference		Reference	
Yes	0.93 (0.82-1.05)	0.25	0.68 (0.59-0.78)	<0.001
Surgery				
No	Reference		Reference	
Yes	0.26 (0.22-0.31)	<0.001	0.32 (0.26-0.39)	<0.001

## Data Availability

Data are available at https://seer.cancer.gov/.

## Supplementary Materials

Supplementary Materials Table S1: nomogram points of the risk factors for DM. Table S2: total points of risk points for predicting DM rate. Table S3: nomogram points of the prognostic factors for OS. Table S4: total points of prognostic factors for predicting 3-year survival probability. Table S5: total points of prognostic factors for predicting 5-year survival probability. Table S6: total points of prognostic factors for predicting 10-year survival probability. Figure S1: online webserver interface of the nomogram for predicting DM. Figure S2: online webserver interface of the nomogram for predicting 3-year OS. Figure S3: online webserver interface of the nomogram for predicting 5-year OS. Figure S4: online webserver interface of the nomogram for predicting 10-year OS.
