# Supplementary material for: Predictors of Distant Metastasis and Prognosis in Newly Diagnosed T1 Intrahepatic Cholangiocarcinoma
Source: Biomed Res Int. 2023 Jan 17;2023:6638755. doi: 10.1155/2023/6638755 (PMC9873431; doi:10.1155/2023/6638755)
Supplement: Supplementary Materials — Table S1: nomogram points of the risk factors for DM. Table S2: total points of risk points for predicting DM rate. Table S3: nomogram points of the prognostic factors for OS. Table S4: total points of prognostic factors for predicting 3-year survival probability. Table S5: total points of prognostic factors for predicting 5-year survival probability. Table S6: total points of prognostic factors for predicting 10-year survival probability. Figure S1: online webserver interface of the nomogram for predicting DM. Figure S2: online webserver interface of the nomogram for predicting 3-year OS. Figure S3: online webserver interface of the nomogram for predicting 5-year OS. Figure S4: online webserver interface of the nomogram for predicting 10-year OS. [file 6638755.f1.docx]

**Table S1 Nomogram points of the risk factors for distant metastasis**

| **Clinicopathological variable**s | **Points** |
| --- | --- |
| Age at diagnosis |  |
| 18-49 | 18 |
| 50-64 | 58 |
| 65-79 | 49 |
| ≥80 | 0 |
| Tumor size |  |
| ≤3cm | 0 |
| >3cm, ≤5cm | 25 |
| >5cm, ≤7cm | 55 |
| >7cm, ≤9cm | 70 |
| >9cm | 100 |
| Unknown | 87 |
| Grade |  |
| I-II | 0 |
| III-IV | 28 |
| Unknown | 50 |
| Lymph node metastasis |  |
| No | 0 |
| Yes | 80 |

**Table S2 Total points of risk points for predicting distant metastasis rate**

| **Total points** | **Predicted distant metastasis rate** |
| --- | --- |
| 12 | 0.05 |
| 70 | 0.10 |
| 133 | 0.20 |
| 174 | 0.30 |
| 208 | 0.40 |
| 240 | 0.50 |
| 271 | 0.60 |
| 305 | 0.70 |

**Table S3 Nomogram points of the prognostic factors for overall survival**

| **Clinicopathological variables** | **Points** |
| --- | --- |
| Age at diagnosis |  |
| 18-49 | 0 |
| 50-64 | 15 |
| 65-79 | 32 |
| ≥80 | 39 |
| Race |  |
| White | 16 |
| Black | 14 |
| Other | 0 |
| Tumor size |  |
| ≤5cm | 0 |
| >5cm | 10 |
| Unknown | 30 |
| Grade |  |
| I-II | 0 |
| III-IV | 11 |
| Unknown | 11 |
| Lymph node metastasis |  |
| No | 0 |
| Yes | 29 |
| Distant metastasis |  |
| No | 0 |
| Yes | 43 |
| Radiation treatment |  |
| None/Unknown | 18 |
| Yes | 0 |
| Chemotherapy |  |
| No/Unknown | 38 |
| Yes | 0 |
| Surgery |  |
| No | 100 |
| Yes | 0 |

**Table S4 Total points of prognostic factors for predicting 3-year survival probability**

| **Total points** | **Predicted 3-year survival probability** |
| --- | --- |
| -3 | 0.85 |
| 23 | 0.8 |
| 60 | 0.7 |
| 89 | 0.6 |
| 113 | 0.5 |
| 135 | 0.4 |
| 157 | 0.3 |
| 180 | 0.2 |
| 209 | 0.1 |

**Table S5 Total points of prognostic factors for predicting 5-year survival probability**

| **Total points** | **Predicted 5-year survival probability** |
| --- | --- |
| -1 | 0.8 |
| 36 | 0.7 |
| 65 | 0.6 |
| 89 | 0.5 |
| 111 | 0.4 |
| 133 | 0.3 |
| 156 | 0.2 |
| 185 | 0.1 |

**Table S6 Total points of prognostic factors for predicting 10-year survival probability**

| **Total points** | **Predicted 10-year survival probability** |
| --- | --- |
| 6 | 0.7 |
| 34 | 0.6 |
| 59 | 0.5 |
| 81 | 0.4 |
| 103 | 0.3 |
| 126 | 0.2 |
| 154 | 0.1 |


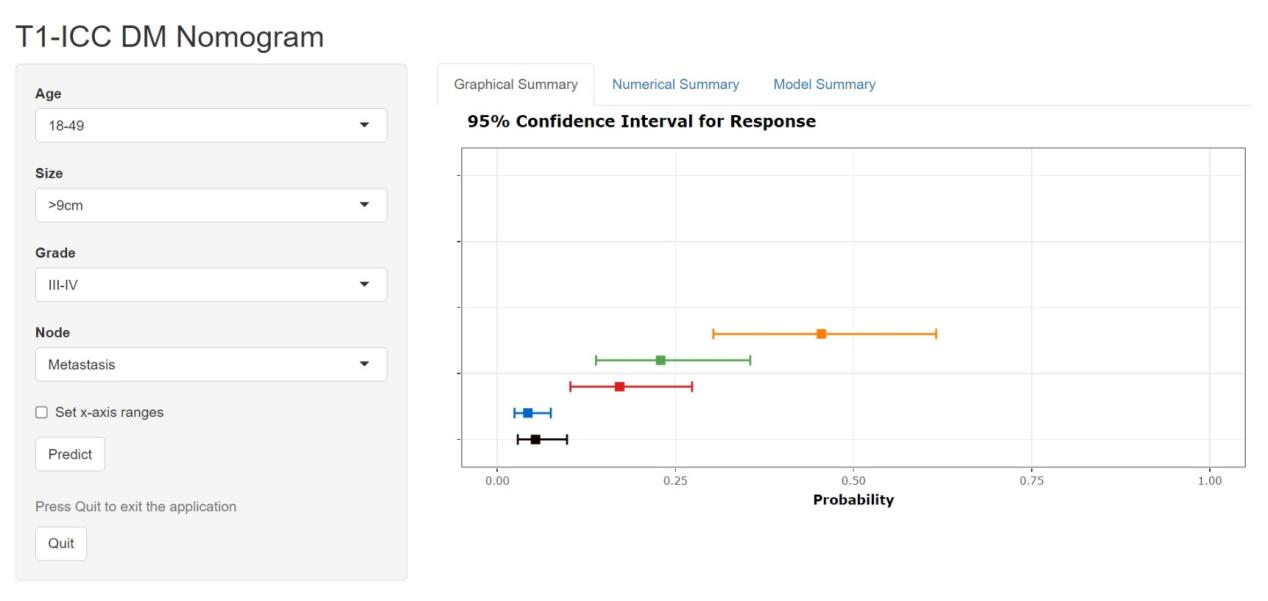


**Figure S1.** Online webserver interface of the nomogram for predicting DM


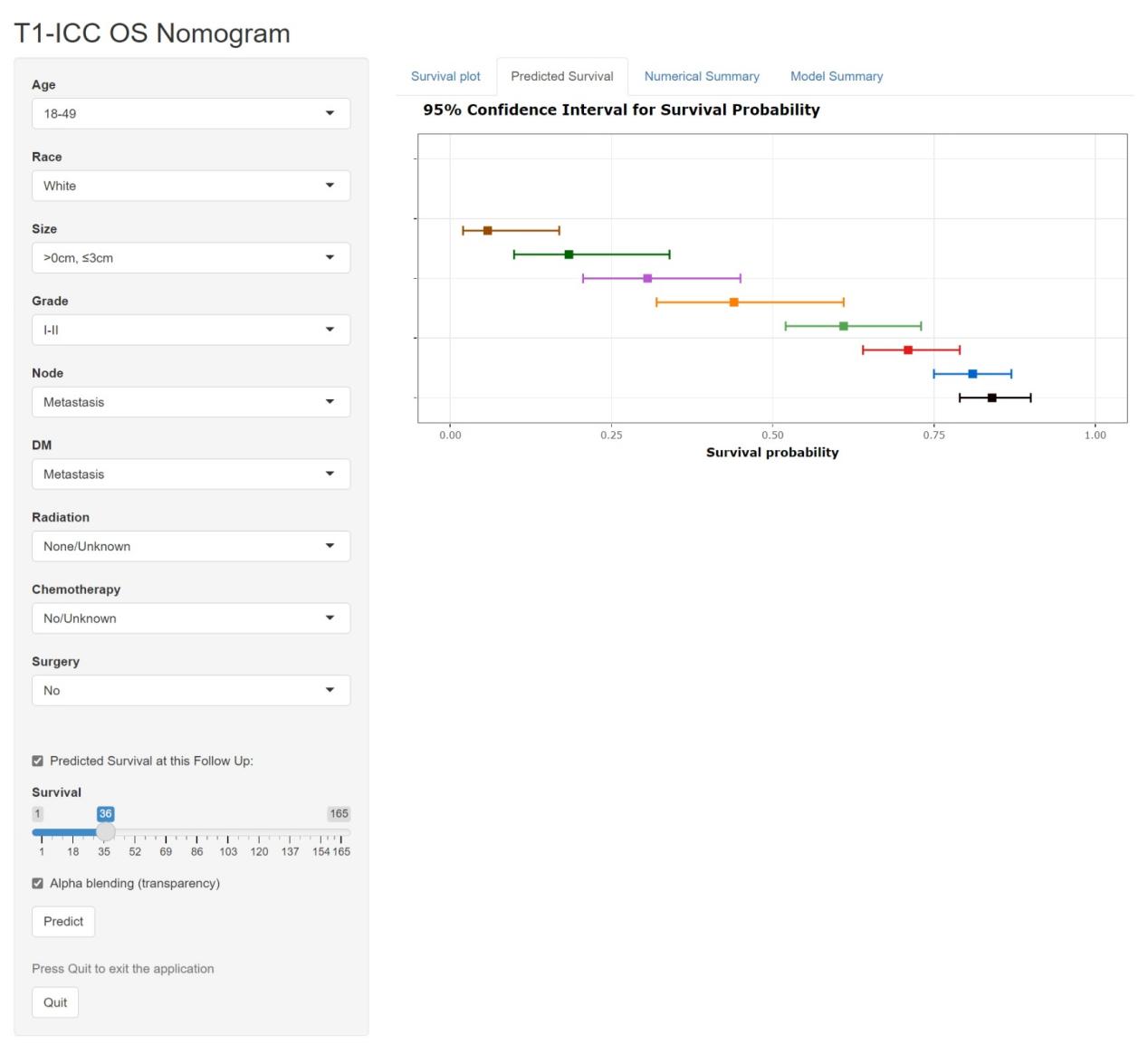


**Figure S2.** Online webserver interface of the nomogram for predicting 3-year OS


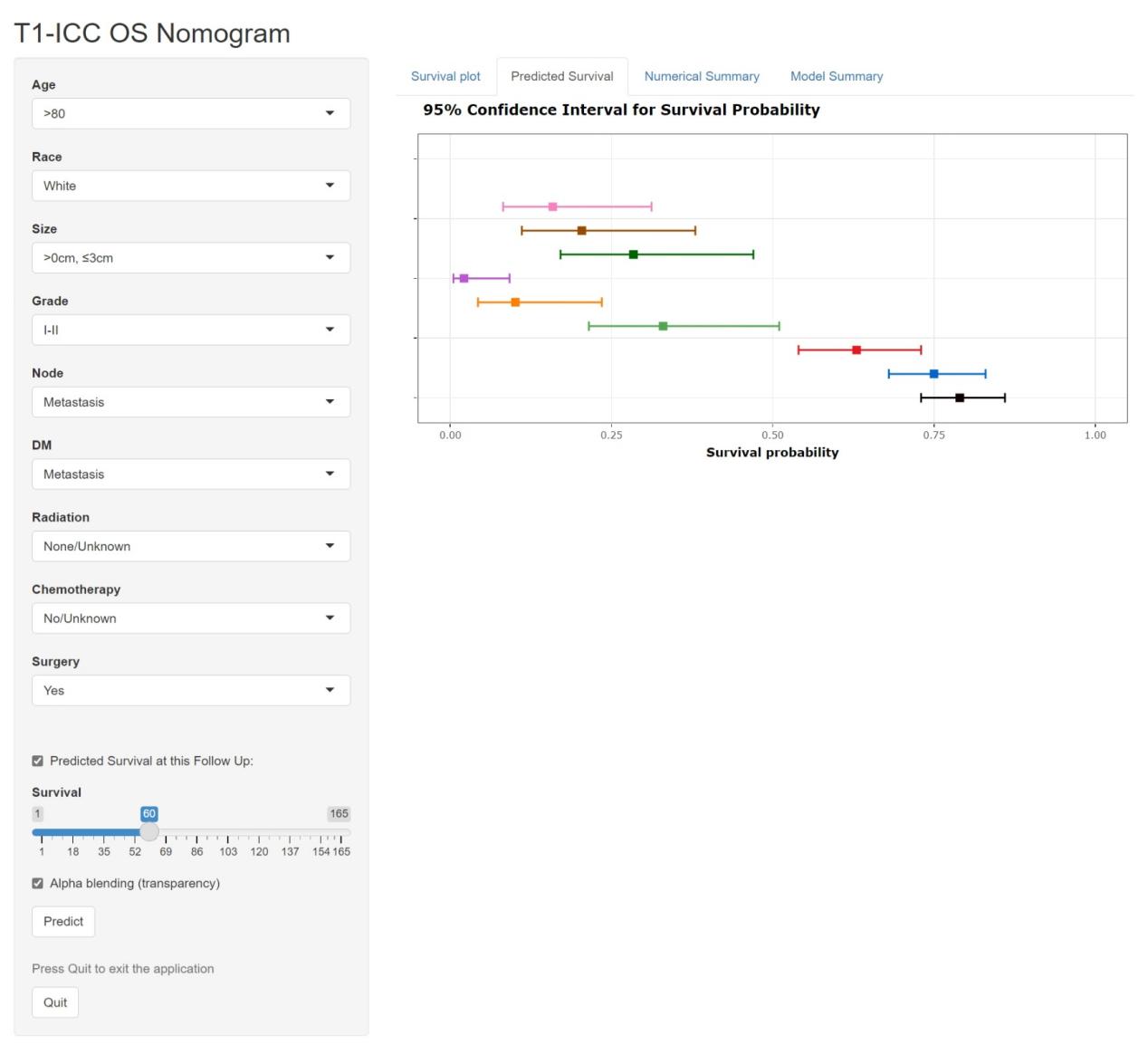


**Figure S3.** Online webserver interface of the nomogram for predicting 5-year OS


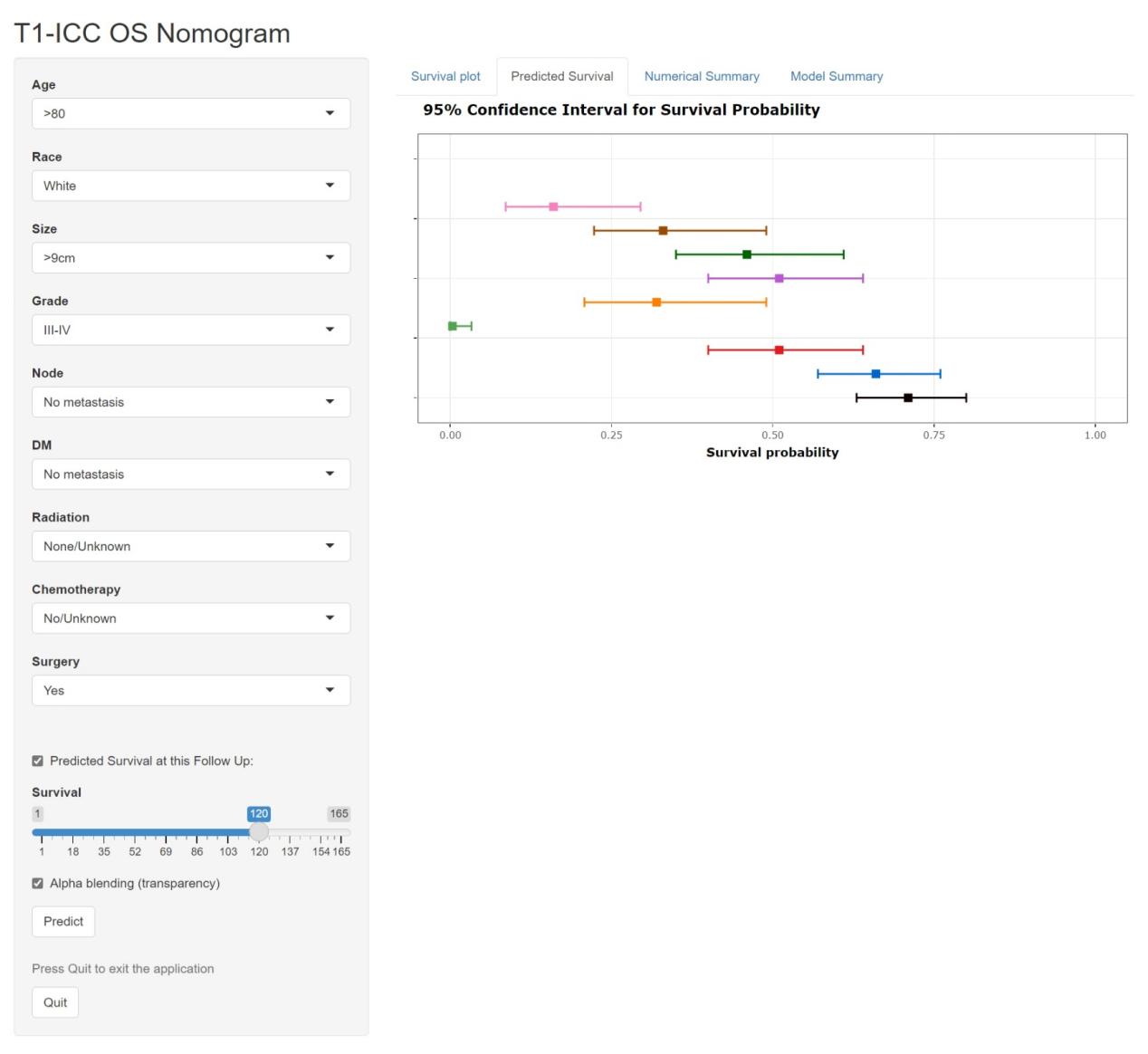


**Figure S4.** Online webserver interface of the nomogram for predicting 10-year OS
